# Haematopoietic stem cell transplantation for cutaneous T‐cell lymphomas: Decision‐guiding prognostic determinants yet to be clarified

**DOI:** 10.1111/jdv.70174

**Published:** 2025-12-23

**Authors:** Marion Wobser

**Affiliations:** ^1^ Department of Dermatology University Hospital Wuerzburg Wuerzburg Germany

Cutaneous T‐cell lymphomas (CTCL) comprise rare and heterogeneous subtypes with variable presentation, biological behaviour and clinical outcome. For advanced stages (≥IIB) of mycosis fungoides (MF) and aggressive subtypes such as Sézary syndrome (SS) response to systemic therapy is often short‐lived and the prognosis is limited. A new prognostic index built up of clinical parameters including age, stage/nodal involvement, elevated LDH and the presence of large cell transformation in the skin was established to better attribute patients with MF and SS to prognostic subgroups and accordingly adjust monitoring and treatment measures.^1^ Therapy for these advanced disease conditions is mostly palliative and innovations are urgently needed to improve not only progression‐free survival but also overall survival (OS). However, the development of targeted and personalized treatment strategies for CTCL is hampered by a high intra‐ and interpatient molecular heterogeneity and divergent clonal evolution of neoplastic cells.^2^

The only curative treatment option for aggressive and advanced CTCL is haematopoietic stem cell transplantation (HSCT). Nevertheless, hitherto published case series and recent national retro‐ and prospective studies on HSCT in CTCL have produced variable results especially with respect to its influence on survival, thus entailing a decision dilemma.^3^

Up to now, clear‐cut prognostic markers governing best timing, ideal patient and donor source selection as well as optimal pre‐ and post‐transplant treatment protocols are lacking. Based on a comprehensive meta‐analysis, reduced‐intensity compared to myeloablative conditioning and complete or at least partial remission of disease before transplantation were associated with improved OS. In addition, the use of total body irradiation/total skin electronic beam therapy was implicated in lower risk of cutaneous relapse/graft‐versus‐host disease.^3^

The publication of Bejarano et al.^4^ in this issue therefore comes at a crucial time adding further real‐world experience to this issue. Based on a large data set of the Primary Cutaneous Lymphoma Registry of the Spanish Academy of Dermatology and Venereology, the authors evaluated the outcome of HSCT in CTCL in a retrospective setting. Propensity score matching for key clinical parameters such as diagnosis, age and stage—adjusting the HSCT versus the non‐HSCT cohort (*n* = 51 per each group)—did not reveal an OS benefit for either group in this analysis.^4^ Although in the HSCT group (*n* = 46 allogeneic HSCT, *n* = 5 autologous HSCT) high response rates were achieved resulting in complete remission in 70.6% of patients, more than half of the transplanted patients relapsed after a median of 14.7 months. Of note, 39.2% of the HSCT group died during the observation period mainly owing to disease progression or HSCT‐associated causes including infectious complications. Influencing factors for the study results may be assigned to the heavily pre‐treated patient cohort with a median of 6.3 lines of prior systemic therapy, the high rate of haploidentical donor source and the disease status before HSCT (complete or partial remission in only 66% of patients).

In contrast, based on updated long‐term results of a prospective, multicentre study (CUTALLO) in France,^5^ propensity score matched analysis of high‐risk and advanced CTCL demonstrated not only significantly better PFS of the allogeneic HSCT cohort (*n* = 55) compared to standard‐of‐care therapy (*n* = 44) but was able to show significantly prolonged OS in transplanted patients.

Once again, both studies illustrate that in the future, it will be an essential task to identify pivotal factors for careful patient selection undergoing HSCT and to optimize conditioning regimens as well as post‐transplant management to enhance survival outcomes. To conclude, as long as innovative treatment modalities fail to induce durable disease remission of CTCL paving the way for a cure of the disease, allogeneic HSCT still represents the only potentially curative treatment option. Therefore, according to international guidelines, this treatment may be offered to CTCL patients upon an individual discussion in an interdisciplinary approach between dermatologists and haematologists.^6^

## FUNDING INFORMATION

None.

## CONFLICT OF INTEREST STATEMENT

Marion Wobser received funding for congress participation and consulting fees by Kyowa Kirin, Takeda, Recordati Rare Diseases and Stemline Therapeutics.

## Data Availability

Data sharing not applicable to this article as no datasets were generated or analysed during the current study.
